# Endoscopic submucosal dissection and overtube-assisted peroral retrieval of a giant duodenal tumor involving the pyloric ring

**DOI:** 10.1055/a-2880-0275

**Published:** 2026-06-03

**Authors:** Takaya Shimura, Makiko Sasaki, Yusuke Okuda, Naomi Sugimura, Mamoru Tanaka, Eiji Kubota, Hiromi Kataoka

**Affiliations:** 1 Department of Gastroenterology and Metabolism 38386 Nagoya City University Graduate School of Medical Sciences and Medical SchoolNagoyaAichi PrefectureJapan

**Fig. 1:**
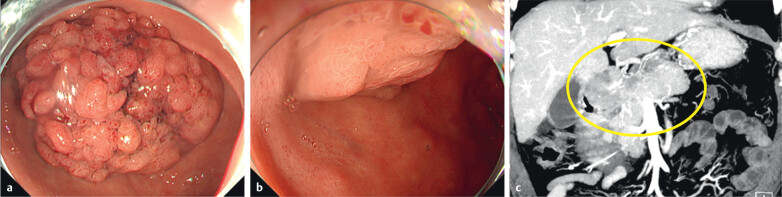
A giant duodenal tumor involving the pyloric ring. ( **a** ) The oral side of the tumor extended beyond the pyloric ring, forming a protruding gastric lesion. ( **b** ) On the anal side of the protruding tumor, a flat lesion extended to the superior duodenal angle. ( **c** ) A contrast-enhanced computed tomography scan revealed a large, highly vascular tumor involving the duodenal bulb and the stomach (yellow circle).

A 60-year-old man complained of periodic postprandial epigastric pain. Esophagogastroduodenoscopy revealed an 80-mm giant, highly vascular duodenal tumor expanding into the stomach ( Fig. 1a–c ). Histopathological examination of the biopsy specimen revealed a high-grade adenoma. The postprandial epigastric pain was thus considered to be due to a ball-valve phenomenon. Although this duodenal adenoma was considered an indication for endoscopic submucosal dissection (ESD), two major challenges were anticipated: performing duodenal ESD on such a giant tumor and retrieving the resected specimen perorally and endoscopically.

**Fig. 2:**
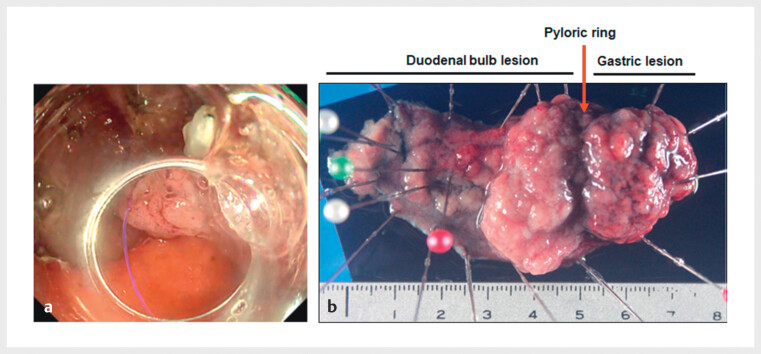
Duodenal ESD. ( **a** ). After submucosal dissection beyond the pyloric ring, a line-attached clip was placed on the oral side of the dissected tissue, providing traction and maintaining a clear visual field. ( **b** ) Pathological diagnosis was high-grade adenoma. The right and left regions show the gastric lesion and the main duodenal lesion, respectively. The central red arrow indicates the pyloric ring. ESD, endoscopic submucosal dissection.

Under general anesthesia, the patient underwent duodenal ESD using a 1.5-mm DualKnife J (Olympus, Tokyo, Japan) and a therapeutic colonoscope (PCFH290TI: Olympus) fitted with a distal attachment (DH-28GR: Fujifilm, Tokyo, Japan). A line-attached clip was applied during ESD. 1​ ​2​ Subsequently, the giant tumor was successfully resected en bloc ( Fig. 2a and b ).

**Fig. 3:**
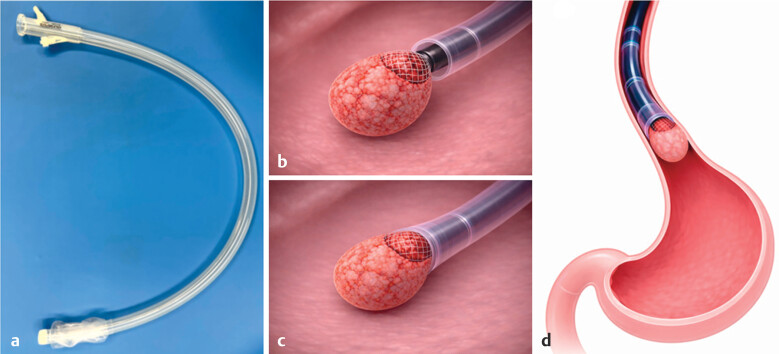
A novel endoscopic tumor retrieval using an overtube. ( **a** ) An overtube for the colonoscope (ST-CB1; Olympus). ( **b–d** ) Schematic illustration of the novel tumor retrieval. The schematic illustration was created using ChatGPT image generation (OpenAI, San Francisco, CA, USA). The tumor was grasped ( **b** ), pulled, and wedged to the overtube ( **c** ) to prevent catching or rotation at the cardia. ( **d)** The integrated endoscope, overtube, and tumor were slowly withdrawn together, finally achieving successful peroral tumor retrieval.

For tumor retrieval, the resected tumor was grasped using a loop retrieval net (MED-132-NET; Meditalia, Palermo, Italy). The large tumor could not successfully pass through the cardia by simply grasping it because of the anatomically stenotic area. To prevent tumor impaction at the cardia during retrieval, we used an overtube (single-use splinting tube for colonoscope, ST-CB1; Olympus). This overtube was inserted beyond the cardia into the stomach. The resected tumor was grasped, wedged and integrated with the overtube, which were slowly withdrawn together ( Fig. 3a–d ). Finally, the tumor was successfully retrieved perorally.

This tumor was pathologically diagnosed as an 80-mm high-grade adenoma ( Fig. 2b ). On postoperative day 8, the patient was discharged without any adverse events. No recurrence was observed thereafter.

In conclusion, a giant duodenal tumor involving the pyloric ring can be successfully resected using a fully noninvasive approach, thereby overcoming the technical challenges of duodenal ESD and peroral tumor retrieval. We demonstrate a novel overtube-assisted endoscopic retrieval technique ( Video 1 ).

**Video 1** Duodenal endoscopic submucosal dissection and overtube-assisted peroral retrieval. The schematic illustration of the overtube-assisted technique was created using ChatGPT image generation (OpenAI).


Endoscopy_UCTN_Code_TTT_1AO_2AG_3AD
